# Physically Cross-Linked PVA Hydrogels as Potential Wound Dressings: How Freezing Conditions and Formulation Composition Define Cryogel Structure and Performance

**DOI:** 10.3390/pharmaceutics16111388

**Published:** 2024-10-28

**Authors:** Anna Górska, Ewelina Baran, Justyna Knapik-Kowalczuk, Joanna Szafraniec-Szczęsny, Marian Paluch, Piotr Kulinowski, Aleksander Mendyk

**Affiliations:** 1 Department of Pharmaceutical Technology and Biopharmaceutics, Faculty of Pharmacy, Jagiellonian University Medical College, 9 Medyczna Street, 30-688 Kraków, Poland; aleksander.mendyk@uj.edu.pl; 2 Institute of Technology, University of the National Education Commission, Podchorążych 2, 30-084 Kraków, Poland; ewelina.baran@uken.krakow.pl (E.B.); piotr.kulinowski@uken.krakow.pl (P.K.); 3 Faculty of Science and Technology, Institute of Physics and SMCEBI, University of Silesia, 75 Pułku Piechoty 1a, 41-500 Chorzów, Poland; justyna.knapik-kowalczuk@smcebi.edu.pl (J.K.-K.); marian.paluch@us.edu.pl (M.P.); 4 CHDE Polska S.A, Biesiadna 7, 35-304 Rzeszow, Poland; joanna.szafraniec@chde.pl

**Keywords:** cryogels, hydrogel wound dressings, polyvinyl alcohol, physical cross-linking

## Abstract

**Objectives**: Hydrogels produced using the freeze–thaw method have demonstrated significant potential for wound management applications. However, their production requires precise control over critical factors including freezing temperature and the choice of matrix-forming excipients, for which no consensus on the optimal conditions currently exists. This study aimed to address this gap by evaluating the effects of the above-mentioned variables on cryogel performance. **Methods**: Mechanical properties, absorption capacity, and microstructure were assessed alongside advanced analyses using differential scanning calorimetry (DSC) and low-field nuclear magnetic resonance relaxometry (LF TD NMR). **Results**: The results demonstrated that fully hydrolyzed polyvinyl alcohol (PVA) with a molecular weight above 61,000 g/mol is essential for producing high-performance cryogels. Among the tested formulations, an 8% (*w*/*w*) PVA_56–98_ solution (Mw~195,000; DH = 98.0–98.8%) with 10% (*w*/*w*) propylene glycol (PG) provided the best balance of stretchability, durability, and low adhesion. Notably, while −25 °C is often used for cryogel preparation, freezing the gel precursor at −80 °C yielded superior results, producing materials with more open, interconnected structures and enhanced mechanical strength and elasticity—deviating from conventional practices. **Conclusions**: The designed cryogel prototypes exhibited functional properties comparable to or even surpassing commercial wound dressings, except for absorption capacity, which remained lower. Despite this, the cryogel prototypes demonstrated potential as wound dressings, particularly for use in dry or minimally exuding wounds. All in all, this study provides a comprehensive analysis of the physicochemical and functional properties of PVA cryogels, establishing a strong foundation for the development of advanced wound dressing systems.

## 1. Introduction

The term cryogel, derived from the Greek κρύος (kryos), meaning cold, refers to a class of hydrogels that are developed under sub-zero temperatures through the repetitive freezing (typically at −20 °C) and thawing (at room temperature) of a solution containing monomeric or polymeric precursors [1,2,3]. The underlying principle of this process is the physical cross-linking of polymer chains, facilitated by the phase transitions of water. During freezing, part of the solvent turns into ice, but a portion remains in a liquid state, known as the “unfrozen liquid microphase”. This microphase is where the dissolved polymers concentrate and undergo cross-linking processes. Upon thawing, the ice melts, allowing the polymer chains to form stable cross-links, thereby solidifying the cryogel structure and ultimately forming a solid hydrogel membrane [4,5,6].

The main established techniques for hydrogel manufacturing, apart from the freeze–thaw method, include the use of chemical crosslinkers (e.g., sodium bicarbonate, glutaraldehyde, glyceraldehyde), high-energy radiation, free-radical polymerization, click reactions (such as copper-catalyzed azide–alkyne cycloaddition), and ionic interactions [7,8,9]. Among these, the freeze–thaw method has several advantages. A primary benefit is its ability to cross-link polymers without using potentially toxic chemical agents, resulting in non-toxic, biocompatible materials that are well-suited for biomedical applications [3,10]. This method also eliminates the additional purification steps typically required in chemical cross-linking methods and avoids the special safety precautions associated with, e.g., radiation methods. Moreover, it reduces environmental impact by minimizing chemical waste, making it a more sustainable alternative. Additionally, the freeze–thaw method is cost-effective, straightforward, and versatile, allowing the production of cryogels in various structures, shapes, and sizes, suitable for applications in many diverse areas [11,12].

The freeze–thaw method offers a promising approach to developing non-toxic, transparent, and soft hydrogel wound dressings with tunable functional properties, such as absorption capacity and mechanical strength [13]. These properties can be modulated by adjusting freezing parameters, including its duration, temperature, the number of freeze–thaw cycles, and the polymer’s molecular weight and concentration [3]. Precise control of these factors is essential to address specific wound healing requirements. However, the tunability of this method also introduces challenges due to the lack of standardized protocols for managing these critical factors. The absence of clear procedural guidelines extends the time required for optimization and complicates the replication and confirmation of results by researchers.

In addition to challenges in material preparation, assessing the functionality of cryogel prototypes for wound care applications also presents several difficulties. This complexity arises from the absence of well-defined test methods that are universally adopted by researchers in this field. Although some evaluation methods exist—such as the EN 13726 series, which represents European standards for the laboratory testing of wound dressings—they are not universally applicable to all types of dressings and do not fully cover the issues related to their usability and functionality. The variability in testing conditions across studies, along with the absence of a consensus on threshold values for key quality parameters, complicates the evaluation of materials and hinders the comparability of results across different research groups.

Among the various materials used in cryogel production, polyvinyl alcohol (PVA) is frequently employed due to its proven effectiveness in forming hydrogels with robust structural integrity [6,14]. Its biodegradability, biocompatibility, and non-toxic nature also make it suitable for use in wound dressing formulations [2,15,16,17], where different grades of PVA—characterized by varying molecular weights and degrees of hydrolysis—are commonly used. These parameters are critical as they significantly impact the mechanical strength, porosity, and absorption capacity of the resulting cryogels—factors that are essential for their effectiveness in wound care applications. However, there is still no consensus on the optimal combination of molecular weight and degree of hydrolysis that would provide the best balance of properties in cryogels intended for wound management. Numerous studies have explored the relationship between these parameters, showing that higher molecular weight generally enhances mechanical strength but often reduces flexibility and porosity. This is because as molecular weight increases, polyvinyl alcohol chains become longer, facilitating the formation of larger crystalline regions. In turn, fully hydrolyzed PVA grades exhibit higher crystallinity and strength but tend to be less soluble compared to partially hydrolyzed variants, which offer better water absorption but reduced mechanical strength [3,9].

All in all, while the potential of cryogels in wound care remains promising, considerable work is still needed to refine and standardize their production and application.

This study aimed to achieve several key objectives: (1) establish a foundational basis for optimizing cryogel manufacturing protocols with properties tailored to wound dressing applications. This includes the selection of an appropriate polymer as the matrix-forming agent and the optimization of freeze temperatures to achieve critical attributes such as structural integrity in the presence of exudate, sufficient mechanical strength, high flexibility, low adhesion, and a slightly acidic pH; (2) analyze the effect of physical crosslinking in PVA cryogels using differential scanning calorimetry (DSC) and nuclear magnetic resonance (NMR). This characterization aimed to reveal how freezing conditions affect water presence and polymer chain packing in PVA-based materials, crucial for their optimization in biomedical applications; and (3) compare prepared hydrogel materials with two commercial hydrogel wound dressings: Product 1, composed of polyvinylpyrrolidone, polyethylene glycol, and agar, and Product 2, consisting of polyurethane, propylene glycol and a reinforcing additional layer—a polyurea membrane for enhanced mechanical strength. These assessments, conducted using identical methodologies, aimed to establish reference values for critical performance parameters (mechanical strength, elasticity, and absorption capacity). The comparison was intended to facilitate the evaluation of the functionality and potential of the newly developed cryogels as effective wound dressings. By addressing these goals, the study provides valuable insights into the properties of cryogels, contributing to the ongoing optimization of these hydrogels and supporting the development of more effective third-generation wound dressings.

## 2. Materials and Methods

### 2.1. Materials

Several grades of polyvinyl alcohol (Mowiol^®^, abbreviated as M in the sample names) supplied by Merck (Warsaw, Poland) were used. The partially hydrolyzed grades included Mowiol 8–88 (Mw~67,000; DH = 87–88%), Mowiol 18–88 (Mw~130,000; DH = 86.7–88.7%), and Mowiol 10–98 (Mw~61,000; DH = 98.0–98.8%). The fully hydrolyzed grades comprised Mowiol 28–99 (Mw~145,000; DH = 99.0–99.8%), Mowiol 56–98 (Mw~195,000; DH = 98.0–98.8%), and Mowiol 20–98 (Mw~125,000; DH = 98.0–98.8%). Propylene glycol, used as a plasticizer, was purchased from Chempur (Piekary Slaskie, Poland). Sodium chloride was obtained from Avantor Performance Materials (Gliwice, Poland). Mucin (from porcine stomach, type II) was purchased from Merck (Warsaw, Poland). All materials utilized in this study were of analytical grade.

### 2.2. Procedure for Hydrogel Wound Dressing Preparation

In the first step, we selected the optimal type of polyvinyl alcohol (PVA) to serve as a matrix for forming hydrogel wound dressings. Initially, hydrogel membranes were prepared using six different grades of PVA, including both partially hydrolyzed and fully hydrolyzed types (Table 1). All hydrogels were composed of 10% (*w*/*w*) PVA, 10% (*w*/*w*) propylene glycol (PG), and 80% (*w*/*w*) distilled water. We adopted a 10% PVA concentration as the starting point for our formulations, based on a literature review, which identified this concentration as optimal for balancing mechanical strength and flexibility—both critical for wound dressing applications [18,19].

The PVA was dissolved in distilled water at 90 °C under slow stirring. The dissolution time of the PVA varied based on its type, ranging from approximately 1.5 to 4 h, with fully hydrolyzed PVA polymers requiring the longest time to dissolve. The most challenging dissolution occurred with PVA_56–98_ (Mw~195,000; DH = 98.0–98.8%), which took up to 4 h for complete dissolution. After cooling, evaporated water and PG were added. The mixture was left at room temperature overnight to remove air bubbles. To achieve the intended solid sheet form in the next step, the prepared PVA solutions were subjected to cyclic freeze–thawing. This process involved six consecutive freeze–thaw cycles, each cycle consisting of freezing at −80 °C for one hour followed by thawing at room temperature for one hour. However, the first freezing step was extended to 24 h to ensure thorough solidification [26], marking the start of the first cycle.

Additionally, for the selected optimal type of PVA, we investigated the effectiveness of various freezing conditions beyond the low-temperature freezer at −80 °C (Nuaire NU-9483E, Biogenet, Józefów, Poland), including (a) temperature freezer at −25 °C (Liebherr, GN 3023, Ruda Śląska, Poland), (b) dry ice (−78 °C), and (c) liquid nitrogen (−196 °C) for an exceptional 6 min freezing. A detailed composition of hydrogel systems is provided in Table 2. All of these formulations were subjected to a physical cross-linking process using four different freezing techniques.

### 2.3. Hydrogel Dressings Characterization Methods

#### 2.3.1. Scanning Electron Microscopy Analysis (SEM)

The surface morphology and porosity of hydrogel wound dressings based on the optimal PVA type were analyzed using a scanning electron microscope (PhenomWorld, Thermo Fisher Scientific, Waltham, MA, USA) equipped with a CeB_6_ electron source and a backscattered electron detector. Prior to imaging, all freshly prepared hydrogel membranes were lyophilized for 48 h (Alpha 2–4/LD, Martin Christ GmbH, Osterode am Harz, Germany). The lyophilized samples were mounted on conductive adhesive tape attached to a holder for non-conductive samples. SEM imaging was conducted at an acceleration voltage of 10 kV and a magnification of 1500×.

Porosity and pore sizes were calculated using the SEM images processed with Fiji software (version 2.15.1) and presented as average values [27]. The percentage of pores was measured in triplicate, while pore length and width were manually measured in 150 locations for each formulation using random sampling across the entire image. For porosity determination, a threshold was applied to the images to define pore boundaries, enabling automated porosity calculations. In contrast, pore sizes were measured manually.

Statistical analysis was performed with R software (v 4.4.1, R Core Team, 2024) via ANOVA and Tukey’s post hoc test. Statistical significance was concluded for *p* < 0.05.

#### 2.3.2. Thermal Analysis

A STARe Differential Scanning Calorimeter (DSC) (Mettler Toledo, Greifensee, Switzerland) was utilized to characterize the thermal properties of hydrogel membranes prepared using different freezing temperatures. The DSC, equipped with an HSS8 ceramic sensor and a liquid nitrogen cooling attachment, was calibrated for temperature and enthalpy using zinc and indium standards. Approximately 30 mg of each sample was placed in 40 µL aluminum crucibles. The thermal cycle included (i) heating from −130 °C to 185 °C at a rate of 10 °C /min, (ii) cooling from 185 °C to −130 °C at a rate of 20 °C /min, and (iii) reheating from −130 °C to 185 °C at a rate of 10 °C /min. To determine residual water content and evaporation rates, samples were heated from −130 °C to 70 °C, annealed at 70 °C for 5 min, weighed, and cooled back to −130 °C. This procedure was repeated until no further mass reduction was observed. Melting temperatures (T_m_) were identified as the onset of endothermic peaks, while glass transition temperatures (T_g_) were determined as the midpoint of the heat capacity increase.

#### 2.3.3. Nuclear Magnetic Resonance Relaxometry

LF TD NMR relaxometry was performed using a 23 MHz NMR Rock Core Analyzer with PROSPA 4.26 software (Magritek, New Zealand and Germany). CPMG and IR-CPMG pulse sequences were used for obtaining T_2_ relaxation times spectra and 2D T_1_–T_2_ relaxation time maps, respectively (repetition time = 5 s and 7 s, echo time = 60 µs, dwell time = 1 µs, number of points = 64,000, pulse length = 12 µs). Data were analyzed using an Inverse Laplace Transform algorithm, with T_2_ distribution obtained via the Lawson–Hanson algorithm and 2D maps via FISTA. Hydrogel membranes were wrapped in low-density polyethylene cling film, with the background signal subtracted. The calculations were made using Prospa 4.26 and OriginPro 2021b (OriginLab Corporation, Northampton, MA, USA).

#### 2.3.4. Assessment of pH

The pH of the polyvinyl alcohol (PVA) solutions prior to freeze–thaw cycling was assessed using a pH meter (Mettler Toledo™ S220, Greifensee, Switzerland). Prior to measurements, the pH meter was calibrated with standard buffer solutions. The pH electrode was then immersed in a beaker containing the PVA solution, and the pH value was recorded. Measurements were performed in triplicate, and the results are expressed as mean values ± standard deviation (SD).

### 2.4. Functional Properties of Cryogels

#### 2.4.1. Water Uptake Capacity

The water uptake capacity (Uw) of hydrogel membranes was determined gravimetrically. Membrane samples (2 cm × 2 cm) were first equilibrated in a constant climate chamber (Memmert HPP 108, Schwabach, Germany) at 25 °C and 60% relative humidity (RH) for 24 h, then weighed (Wd) and immersed in a 0.9% NaCl solution at 32 °C approximating skin temperature. At specific time intervals of 10, 20, and 30 min and 1, 2, 3, 4, 5, 6, 7, 8, and 24 h, respectively, samples were removed from the solution, gently blotted with filter paper to remove excess solution, reweighed (Ws), and re-submerged into the solution. The average water uptake was calculated as a percentage (*n* = 6) ± standard deviation (SD) using Equation (1).

Uw = (Ws − Wd)/Wd·100%(1)

where Ws is the weight of the swollen sample [g], and Wd is the initial weight of the sample [g].

#### 2.4.2. Mechanical Properties

Mechanical properties were assessed using a mechanical testing machine (Shimadzu EZ-SX, Kyoto, Japan) equipped with a 20 N load cell. Prior to testing, samples were conditioned in an HPP 108 climate chamber at 25 °C and 60% RH. Two methods were employed, both involving tensile testing:

Test 1:

Paddle-shaped specimens (75 mm long, 10 mm wide at the ends, and 5 mm wide in the middle) were mounted in the machine’s grips and subjected to uniaxial tensile testing at a strain rate of 1000 mm/min until failure. Each test was repeated 6 times. For a detailed illustration of the experimental setup and procedure, refer to our earlier article [28].

Statistical analysis was performed with R software (v 4.4.1) via ANOVA and Tukey’s post hoc test. Statistical significance was concluded for *p* < 0.05.

Test 2:

Rectangular specimens (2.5 cm × 14 cm) were prepared according to the EN 13726-4:2003 standard, marked with parallel lines 10 cm apart. The samples were mounted in the machine grips and stretched to 20% elongation at 300 mm/min. After holding the stretch for 60 s, the specimens were removed and allowed to relax for 300 s. Permanent deformation was then calculated following the standard’s guidelines:

(2) $$e\left[\%\right]=\frac{\left(L_{2}-L_{1}\right)}{L₁}\times 100\%$$

where e was the permanent deformation [%], L_1_ was the initial length [cm], and L_2_; was the length after stretching and relaxation [cm].

The test for each formulation was repeated 3 times.

#### 2.4.3. Adhesive Properties

Adhesive properties were tested using a mechanical testing machine (Shimadzu EZ-SX, Kyoto, Japan). A mucin disc (10 mm diameter, 365 mg) soaked in a 10% (*w*/*w*) mucin solution served as a model for natural mucin. The disc was attached with double-sided tape to a flat measuring probe (11 mm diameter), which was pre-wetted with the mucin solution for 30 s. The hydrogel membrane sample was placed on a stationary table, and a 0.1 N force was applied for 1 min. Afterward, the probe was withdrawn at 1 mm/s. The adhesive strength was quantified as the peak force recorded during the detachment of the mucin disc from the membrane. Each test was repeated three times for accuracy. For a detailed illustration of the experimental setup and procedure, refer to our earlier article [28].

## 3. Results and Discussion

### 3.1. Preliminary Insights on Polymer Selection for Wound Dressing Applications

According to the generally accepted requirements, an “ideal” wound dressing should demonstrate the following characteristics: (1) structural integrity in contact with exudate: ensures easier removal of the dressing from the wound bed, reducing the risk of secondary infection from unremoved fragments; (2) low adhesion: minimizes the risk of damage to newly formed tissues during dressing removal; (3) slightly acidic pH: supports skin barrier functions and prevents microbial colonization; (4) high flexibility: allows freedom of movement and adaptation to body shape, ensuring that the dressing covers the wound bed tightly without leaving air pockets; and (5) sufficient mechanical strength: provides resistance to stretching and stresses exerted by the body, enhances cushioning capabilities, and ensures that the dressing can withstand handling and application without tearing [14,29,30,31].

To develop effective wound dressings, it is crucial to integrate key physicochemical properties with the careful selection of excipients that meet certain critical criteria, including biocompatibility, biodegradability, non-toxicity, and good mechanical strength. Our objective was to develop a simple hydrogel system composed of three primary components: polyvinyl alcohol as the matrix-forming agent, propylene glycol as a plasticizer and humectant to enhance flexibility and prevent drying, and water to improve softness and wound tolerance and provide cooling properties. By focusing on these core components, we aimed to design a material with the potential to be non-irritating, comfortable for use, and cost-efficient to manufacture. Although these components were selected based on their well-established properties that suggest suitability for wound dressings, further biological evaluation is required to confirm their performance in clinical applications. This deliberate simplification of the formulation also allowed us to gain clearer insights into the relationships between formulation variables, particularly the influence of PVA type and cross-linking conditions on the cryogel’s properties.

In the first part of the experiment, different grades of PVA were tested (Table 1). Each hydrogel contained 10% (*w*/*w*) PVA, 10% (*w*/*w*) PG, and 80% (*w*/*w*) water.

At first, the solutions were tested for pH using a calibrated pH meter at room temperature, revealing that all PVA solutions exhibited a slightly acidic pH. This finding corresponds with previous studies, which indicate that an acidic environment can promote wound healing. Specifically, an acidic pH is known to inhibit bacterial growth, support fibroblast migration, proliferation, and growth, and facilitate the release of oxygen from hemoglobin, which is crucial for collagen synthesis and new tissue formation [32,33,34,35,36]. The pH values varied depending on the type of polymer used, ranging from 5.26 to 6.23. The solutions based on PVA_56–98_ (Mw~195,000 g/mol; DH = 98.0–98.8%) and PVA_20–98_ (Mw~125,000; DH = 98.0–98.8%) exhibited the lowest pH among all tested samples. In contrast, samples formulated with PVA_28–99_ (Mw~145,000 g/mol; DH = 99.0–99.8%) demonstrated the highest pH values. Detailed results are presented in Table 3.

Visual assessment revealed no undissolved polymer clusters, and all solutions were easily poured into molds, demonstrating uniformity in dispersion and solubility across all PVA types. The degree of hydrolysis and the molecular weight of polyvinyl alcohol were shown to significantly influence the ability to transform hydrogel systems from a semi-solid to a solid sheet form (Figure S1 in ESI). It was observed that aqueous solutions of partially hydrolyzed PVAs, specifically PVA_8–88_ and PVA_18–88_, did not undergo physical cross-linking during the freeze–thaw process (−80 °C/+22 °C) and remained in a solution state, regardless of their molecular weight.

In contrast, solutions prepared with PVA_20–98_, a fully hydrolyzed PVA with a molecular weight comparable to PVA_18–88_, successfully cross-linked and transformed into a compact form under the same conditions. Similarly, other fully hydrolyzed PVAs, i.e., PVA_56–98_ (Mw~195,000) and PVA_28–99_ (Mw~145,000), also successfully produced soft solid sheets. These materials conformed easily to the shape of the hand and provided a cooling effect upon application.

Interestingly, PVA_10–98_, despite being fully hydrolyzed, failed to achieve adequate cross-linking under the same conditions. Its molecular weight, however, was significantly lower (~61,000) compared to the other fully hydrolyzed grades used in this study. Although solid sheets were formed from 10% PVA_10–98_ solutions, these sheets exhibited very low mechanical strength and disintegrated upon handling (even forceps could not lift the cryogel without causing damage), making further examination impractical. Lower concentrations of PVA_10–98_ did not form solid sheets at all, confirming that this specific PVA grade is unsuitable for the intended application.

Consequently, it was determined that fully hydrolyzed PVA with a molecular weight exceeding 61,000 g/mol is necessary for effective solid sheet formation using the freeze–thaw method. This finding provides a guideline for the selection of polymers when developing hydrogel wound dressings using the cyclic freeze–thaw method.

As expected, all cryogels exhibited low water absorption capacity, with the PVA_28–99_-based material (Mw~145,000; DH = 99.0–99.8%) demonstrating the lowest value (Table 3). However, this characteristic does not preclude their suitability for wound care, as different types of wounds require dressings tailored to specific exudate management needs. The goal of the absorption capacity test was to assess the material’s potential for various wound types. Our results suggest that the designed cryogels may be suitable for use with dry or minimally exuding wounds, aligning with the function of hydrogel dressings currently used in clinical practice [31,37].

One of the key principles in wound care is that wound dressings should be changed as frequently as required but as infrequently as possible to avoid unnecessary disruption to the healing process [38]. For hydrogel wound dressings in solid sheet form, the recommended interval for dressing changes is approximately every 2–3 days, depending on the clinical condition of the wound [31]. A potential issue in wound management is the risk of dressings adhering to the wound bed, which can cause further tissue damage during dressing changes [39]. As presented in Table 3, all cryogels demonstrated low adhesion force values, ranging from 0.099 N (PVA_56–98_ Mw~195,000; DH = 98.0–98.8%) to 0.220 N (PVA_20–98_, Mw~125,000; DH = 98.0–98.8%). These findings are consistent with palpation tests, indicating that the materials, regardless of PVA grade, exhibit weak attachment to the wound bed. This low adhesion facilitates easy dressing removal without disturbing the newly formed epidermis, thus minimizing the risk of tissue damage and enhancing patient comfort during dressing changes.

As shown in Table 4, all cryogels demonstrated high elasticity, with elongation capabilities ranging from approximately 2 to 3 times their original length. The maximum elongation at break varied between 239% to 349%, depending on the polyvinyl alcohol grade used. Notably, when stretched by 20% of their length, all membranes returned to their original shape (e = 0%), indicating shape memory properties. This behavior is a common feature of cryogels, explained by their unique microporous network structure [40,41]. Among the tested materials, the membrane based on PVA_56–98_ demonstrated the highest elongation at break (ɛ = 349%) and exhibited the greatest stretchability. This superior adaptability to dynamic body movements was further supported by its low Young’s modulus (E = 0.03 MPa). In contrast, the membrane based on PVA_28–99_ demonstrated the lowest elasticity. Furthermore, tensile tests confirmed the cryogel’s resistance to accidental tearing, with rupture forces ranging from 2.0 N (PVA_56–98_) to 3.5 N (PVA_28–99_). In contrast, the membrane formulated from PVA_20–98_ exhibited significantly lower mechanical durability, requiring up to six times less force to induce tearing and showing poor dependence under stress. This makes it less suitable for practical applications.

Considering all performance parameters, PVA_56–98_ (Mw~195,000; DH = 98.0–98.8%) was selected as the optimal hydrogel matrix former as it represents the best compromise, offering superior stretchability, durability, the lowest adhesion, and a slightly acidic pH despite its limitation in water absorption. Consequently, PVA_56–98_, was chosen for all further investigations. Nevertheless, the use of PVA_20–98_ also remains promising, and with further optimization of the formulation’s composition, its potential will be explored in our future studies.

### 3.2. Effect of Freezing Conditions and PVA_56–98_ Concentration on Cryogel Properties

#### 3.2.1. Impact of Formulation on pH of Gel Precursors

Healthy human skin generally maintains a pH range of 4 to 6. However, chronic wounds often lead to an increase in pH, rising to levels between 7.3 and 8.9. Research indicates that an alkaline environment can facilitate the proliferation of pathogenic microorganisms. Therefore, using specialized wound dressings capable of modulating the pH of the wound bed may help to accelerate the healing process [36,42].

In this study, the pH of hydrogels based on PVA_56–98_ was measured prior to the freezing–thawing process, with results presented in Table S1 in ESI. All formulations exhibited a pH below 6, thereby meeting the required criteria. Additionally, the incorporation of propylene glycol demonstrated a minor effect on reducing the pH values. Notably, the lowest pH values were observed in samples containing a 10% (*w*/*w*) concentration of propylene glycol.

#### 3.2.2. Visual and Structural Characterization

**Figure 1:**
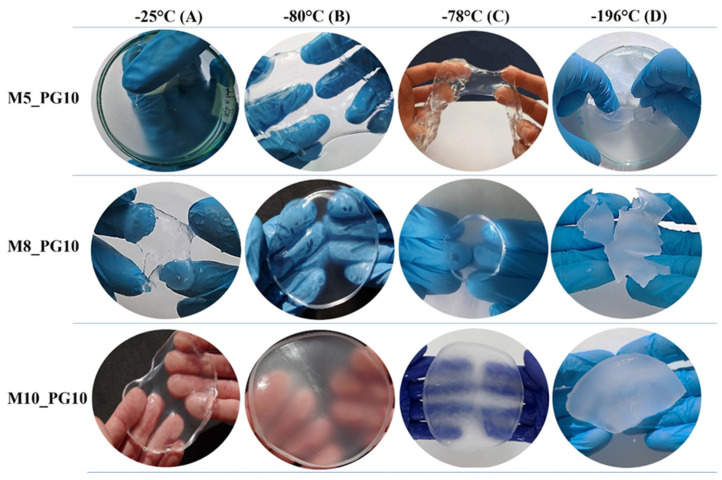
Visual appearance of hydrogel membranes prepared with different PVA concentrations (5%, 8%, 10%) and freezing methods, showing structural differences due to varying preparation conditions.

The selected PVA grade, PVA_56–98_, which showed the most promising properties for hydrogel wound dressing formation, was used to prepare cryogels at various freezing temperatures and PVA concentrations. The impact of both the freezing technique and PVA concentration on the transparency and structural integrity of the cryogels is clearly shown in Figure 1.

Cryogels formed by freezing the solution at −25 °C (laboratory freezer) were transparent with a smooth surface but tended to rupture during palpation. Additionally, reducing the PVA concentration to 5% (*w*/*w*) under these conditions failed to produce a solid sheet. In contrast, solutions frozen at −80 °C (low-temperature freezer) consistently transformed into compact form across all polymer concentrations. This temperature is more versatile, as it reliably produces solid sheets even at lower PVA concentrations. These cryogels exhibited slight opalescence, particularly at 10% (*w*/*w*) PVA, and demonstrated flexibility, conforming well to body contours.

Cryogels formed at −78 °C (dry ice) had a more heterogeneous surface and resisted rupture, like those formed at −80 °C (low-temperature freezer), but displayed greater opalescence. However, the 5% (*w*/*w*) PVA formulation in this group was fragile and prone to rupture. Cryogels prepared by freezing in liquid nitrogen (−196 °C) had the lowest transparency and were extremely fragile. The rapid freezing process under these conditions introduced significant challenges during manufacturing, making it difficult to form intact membranes.

**Figure 2:**
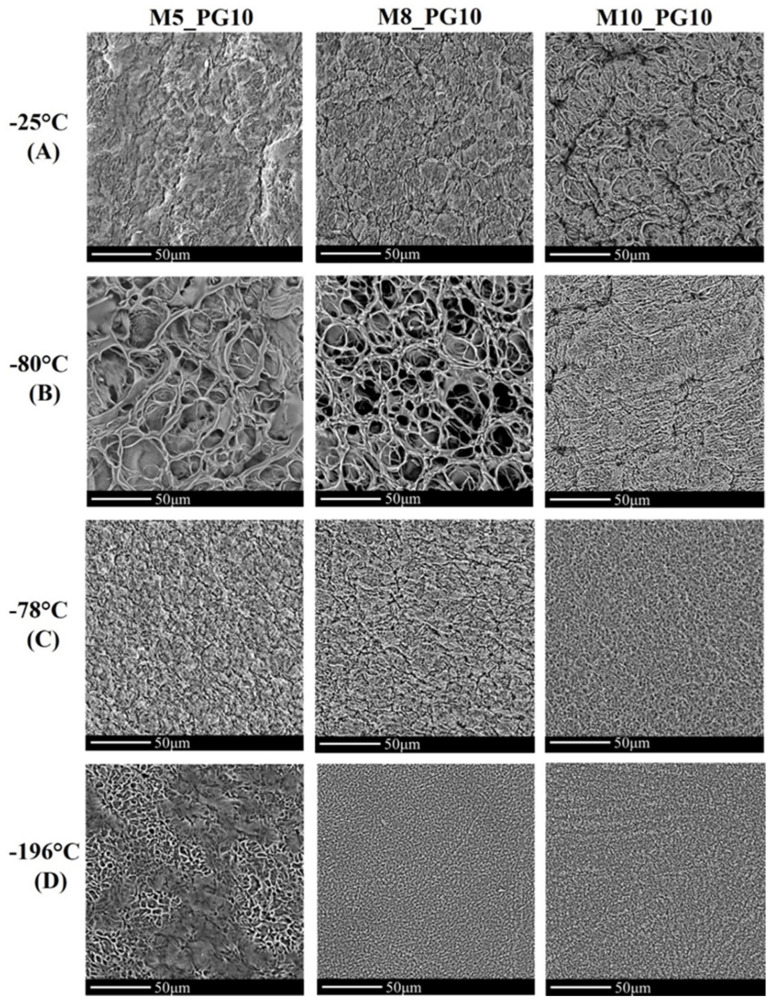
SEM images of hydrogel dressings prepared with varying PVA_56–98_ concentrations and freezing temperatures, captured after 48 h of lyophilization at 1500× magnification.

We further investigated the microstructure and porosity of cryogels using scanning electron microscopy (SEM) following lyophilization. The resulting SEM images (Figure 2) revealed that xerogels (freeze-dried cryogels) exhibited varying degrees of porosity, with denser structures at certain temperatures and more open, interconnected structures at others. The observed morphology appears to be influenced by both PVA crystallization and the dynamics of ice crystal formation during the freeze–thaw process, as explained in previous studies [18,43].

Notably, as shown in Figure 2, the freezing temperature had a substantial impact on the hydrogel microstructure. Contrary to expectations, samples frozen at −80 °C exhibited a more open and interconnected structure with larger, well-defined pores compared to those frozen at −25 °C. This interconnected network, characteristic of cryogels [41], could enhance fluid and gas exchange within the material, a feature potentially beneficial for wound care applications. Although slower freezing at −25 °C was expected to generate larger pores, the samples exhibited a denser, less interconnected network. This unexpected result may be due to specific thermal dynamics, where freezing at −80 °C allowed for better control of ice crystal formation, achieving a better balance between ice crystal growth and the polymer network development throughout the material.

Interestingly, despite the similar freezing temperatures of −80 °C (low-temperature freezer) and −78 °C (dry ice), no similarity was observed in the surface morphology of the xerogels obtained under these conditions. Dry ice effectively transformed the gel precursors into a compact solid sheet; however, it produced smaller pore sizes, potentially limiting the cryogel’s effectiveness as a wound dressing, where larger pores are generally more advantageous for fluid absorption and oxygenation. This confirms that factors beyond just the freezing temperature, such as the rate of freezing, may also influence the final microstructure of the cryogels. In turn, rapid freezing at −196 °C using liquid nitrogen produced a denser structure with smaller and more uniform pores. This effect is likely due to the rapid formation of smaller ice crystals, which occurs because of the faster cooling rate.

Overall, freezing the gel precursor at −80 °C in a laboratory freezer proved to be the most effective method for producing the porous structure characteristic of high-quality cryogels, outperforming other freezing techniques in this field. Consequently, only cryogels formed at −80 °C were selected for further microstructural analysis. The results are presented in Table 5. The data indicated that increasing the PVA concentration led to smaller pores, with pore widths ranging from 1.5 to 13.8 µm and pore lengths between 4.4 and 28 µm. Xerogels formed with 5% PVA exhibited the largest pores, while those with 10% PVA had the smallest. These findings align with the SEM analyses reported by Giusti et al. [37], which demonstrated that both higher concentrations and an increased number of freeze–thaw cycles reduced pore size.

The porosity of the xerogels ranged from 29% to 49%, indicating that xerogels formed with less than 10% (*w*/*w*) PVA had a relatively open structure. This characteristic is advantageous for practical applications such as wound dressings, as it can improve fluid management, comfort, and gas exchange. In the case of pore width and length, all formulations were found to be significantly different between themselves. In the case of porosity, the differences between M5_PG10_B and M8_PG10_B were not significant (*p* = 0.8290).

All in all, these findings underscore the complex interplay between freezing conditions, PVA concentration, and pore formation, highlighting the need for precise control of these parameters to tailor cryogel properties for specific applications.

#### 3.2.3. Water Absorption Capacity

**Figure 3:**
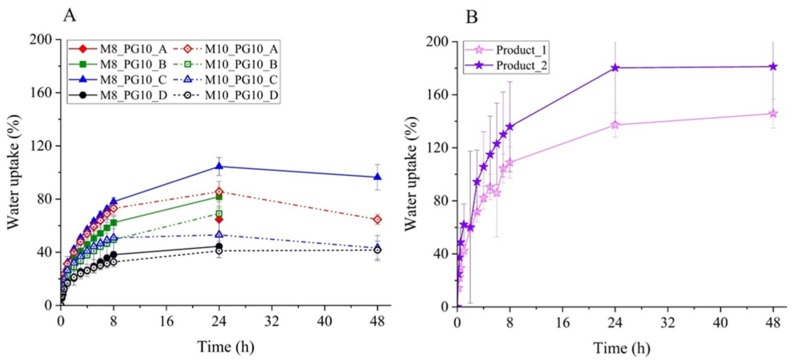
Comparative water uptake (Uw) of lab-developed (**panel A**) and commercial hydrogel wound dressings (**panel B**). Panel A shows the effect of cryogel composition (8% and 10% PVA_56–98_) and preparation method (−25 °C (A), −80 °C (B), −78 °C (C), and −196 °C (D)) on Uw capacity.

Hydrogel dressings, due to their high water content (up to 96%) [31], exhibit limited absorption capacity (from 10% of their equivalent weight) [44], making them most appropriate for dry or minimally exuding wounds. To determine the suitability of designed cryogels for specific wound types, gravimetric tests were conducted, and their absorption capacity was calculated. As shown in Figure 3 and detailed in Table S1 (in the ESI), the results confirmed that the cryogels had a limited absorption capacity, with lower average values compared to selected commercial hydrogel wound dressings. The maximum absorption capacity was achieved after 24 h of incubation in 0.9% NaCl solution and ranged from 41% to 105%, depending on the formulation and freezing conditions. In contrast, commercial dressings, Product 1 and Product 2, exhibited absorption capacities of 137% and 180%, respectively (Figure 3).

An increase in PVA_56–98_ concentration was generally observed to reduce absorption capacity, consistent with SEM analysis (Figure 2, Table 5), which revealed smaller pore sizes at higher PVA_56–98_ concentrations. Freezing temperature also had a significant impact. The cryogel with 8% PVA, frozen at −78 °C (dry ice), showed the highest absorption capacity, while the same formulation frozen at −80 °C had 20% lower absorption, but still ranked second. The cryogel frozen at −196 °C exhibited the lowest absorption capacity.

The cryogels prepared with 5% PVA_56–98_ presented significant challenges during absorption tests. Upon exposure to 0.9% NaCl solution, these samples lost structural integrity and adhered to the filter paper. To mitigate inaccuracies, measurements were taken at a single time point (after 24 h). This finding, combined with the limited water uptake (Table S1 in ESI), suggests that a 5% PVA_56–98_ concentration is insufficient for structural stability, indicating that higher concentrations are needed to ensure the mechanical stability of hydrogels produced via cyclic freeze–thaw methods. Mechanical strength tests (Table S2 in ESI) supported these findings, aligning with the microstructure analysis from SEM (Figure 2).

Similar challenges occurred with cryogels prepared using an 8% PVA_56–98_ solution and more than 5% propylene glycol, frozen at −25 °C. These cryogels also showed poor structural integrity, with absorption data only available after 24 h. Tensile tests confirmed that freezing at −25 °C did not promote sufficient cross-linking, even at an 8% PVA concentration, contrasting with other studies that often use −25 °C as a standard freezing temperature for cryogels.

These findings suggest that the developed cryogels are suitable for dry or minimally exuding wounds. However, further optimization, including the incorporation of polymeric excipients such as sodium alginate or polyvinylpyrrolidone, may improve water absorption and expand their use for moderately exuding wounds. Except for the noted exceptions, all cryogels demonstrated consistent structural integrity upon exposure to 0.9% NaCl solution, suggesting that they are likely to maintain stability after contact with potential exudates.

#### 3.2.4. Adhesive Properties of Cryogels

The adhesion force values for our materials ranged from 0.032 N ± 0.007 N to 0.124 N ± 0.020 N (Table S1 in ESI), placing them between two commercial non-adhesive dressings, which required forces of 0.045 N ± 0.009 N (Product 1) and 0.191 N ± 0.035 N (Product 2) for detachment of the mucin tablet from the dressing sample. This comparison, along with palpation tests, confirms the low adhesion of our materials. While their low adhesion is advantageous for easy removal and gentle wound care, it necessitates the use of secondary dressings such as saline-soaked gauze or polyurethane film to secure them in place and prevent desiccation. However, this is a typical characteristic of hydrogel wound dressings and not a limitation [31], as the benefits of minimal tissue trauma during removal outweigh the need for additional support.

#### 3.2.5. Mechanical Properties of Cryogels

The mechanical properties of hydrogel wound dressings are crucial for evaluating their resistance to tearing under stress and their ability to conform to the treatment area. To assess these properties, tensile testing was conducted. Hydrogel wound dressings are known for their relatively low mechanical strength, which can result in unintentional tearing during handling and application—a notable drawback of many commercial hydrogel wound dressings. Therefore, enhancing their mechanical strength is important for improving their functionality in medical applications.

**Figure 4:**
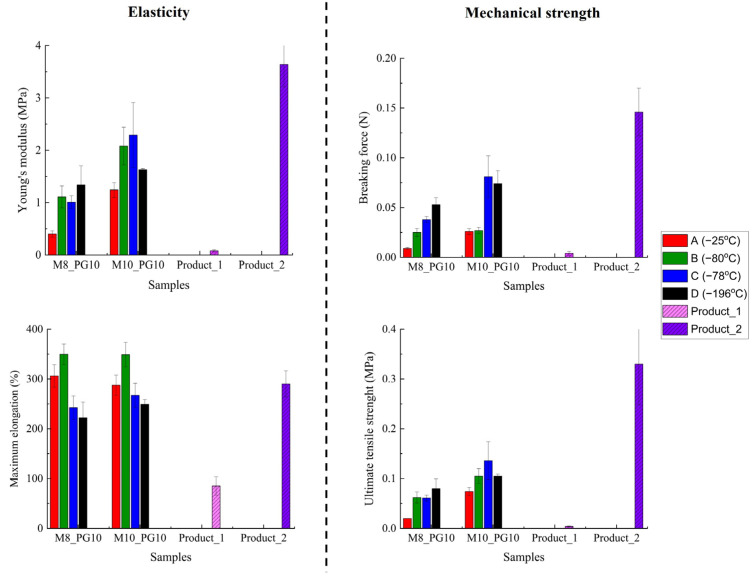
Comparative analysis of mechanical properties: lab-developed dressings (M8_PG10, M10_PG10) vs. commercial hydrogel wound dressings (Product_1, Product_2)—the impact of composition and freezing conditions on elasticity and mechanical strength.

In this study, we examined the effects of freezing conditions and PVA_56–98_ concentration on the mechanical properties of cryogels. Detailed results are presented in Table S2 (ESI). Given the absence of standardized threshold values for mechanical parameters, Figure 4 compares the tensile test results of the designed cryogels with two commercial hydrogel wound dressings, helping to interpret their mechanical performance in relation to the reference products.

The designed cryogels demonstrated high resistance to rupture, with tensile strength (σ) and maximum breaking force (Fmax) significantly exceeding those of Product 1, reaching up to 25 times its breaking strength. Product 2, reinforced with a polyurea membrane, showed greater mechanical strength but at the cost of increased stiffness and reduced elasticity, with a Young’s modulus up to 16 times higher than the designed cryogels. Our materials achieved a balance between strength and flexibility, positioning them as a favorable alternative, especially considering that Product 2 has additional reinforcement.

As expected, increasing the PVA concentration resulted in higher tensile strength and maximum breaking force, consistent with microstructural observations (Figure 2, Table 5) that revealed a progressive reduction in pore size (M5 > M8 > M10). This can be attributed to the higher concentration of hydroxyl groups, which promote stronger intermolecular hydrogen bonding, contributing to the material’s overall structural integrity. This phenomenon is supported by previous findings, such as those reported by Lozinsky et al. [45], where increased hydrogen bonding resulted in denser, less porous network structures in PVA-based materials.

Cryogels formed at −176 °C and −78 °C, which had denser structures (Figure 2), showed the highest tensile strength but also increased stiffness. In contrast, cryogels formed at −25 °C and −80 °C demonstrated greater elasticity, which is an important factor for wound dressing applications. With low Young’s modulus values of up to 0.027 MPa and maximum elongation ranging from 287% to 349%, these cryogels demonstrated significant elasticity. Notably, cryogels formed at −80 °C, in particular, showed superior stretchability compared to commercial dressings, indicating their potential suitability for wound management. The high elasticity observed suggests that these materials conform well to body contours. These properties could potentially improve patient comfort and support better healing by maintaining consistent contact with the wound.

For the parameters of ultimate tensile strength and breaking force, most of the differences were found to be significant, whereas for Young’s modulus and maximum elongation at breaking, this tendency was reversed and most of the differences were found to be non-significant (Tables S3–S6, ESI).

Additionally, our results (Table S2, ESI) demonstrated that increasing concentrations of propylene glycol as a plasticizer improved the elasticity of the cryogels, with a 10% (*w*/*w*) PG concentration significantly enhancing their stretchability, suggesting improving adaptability to body movements.

Based on overall performance, cryogels formed at −80 °C were identified as the most practical and effective option. This freezing method offers greater control over the cross-linking process, resulting in open, interconnected structures with sufficient mechanical strength, higher stretchability, and reliable structural integrity when exposed to potential exudates. In contrast, cryogels formed at −78 °C (dry ice) demonstrated better absorption capacity and slightly higher mechanical strength. However, their denser structure, increased stiffness, and challenges in controlling the cross-linking process make freezing at −78 °C less suitable for manufacturing hydrogel dressings compared to the −80 °C method. Among the various PVA concentrations tested, 8% was found to be optimal, providing a balance of adequate mechanical strength, high flexibility, and low adhesion compared to 5% (*w*/*w*) or 10% (*w*/*w*) concentrations.

In conclusion, the use of 8% (*w*/*w*) PVA_56–98_ combined with cryogel formation at −80 °C represents the best compromise for potential wound dressing applications, offering adaptability, strength, and flexibility.

### 3.3. Mechanistic Insights into Cryogel Properties through Thermal and Relaxometric Techniques

To further explain and support the observed variations in the functional properties of the cryogels described above, we conducted additional analyses using differential scanning calorimetry (DSC) and nuclear magnetic resonance relaxometry (LF TD NMR). These techniques were employed to provide deeper insights into the designed cryogels and present a more detailed explanation of how differences in cross-linking density, influenced by variations in freezing conditions and polymer concentration, affect the mechanical and structural characteristics of the cryogels.

#### 3.3.1. Thermal Analysis

**Figure 5:**
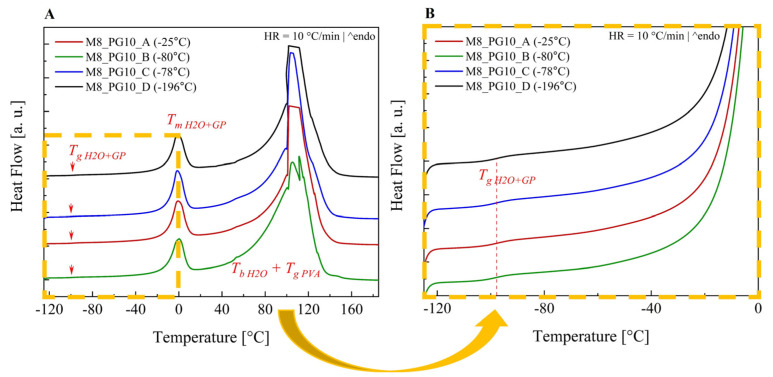
Thermograms of 8% (*w*/*w*) PVA_56–98_ cryogels formed at various freezing temperatures: (**A**) full range and (**B**) magnified region from −120 °C to 0 °C.

Regardless of the preparation methods (freezing temperature), the DSC curves of M8 cryogels registered during heating with a rate of 10 K/min (Figure 5A) revealed three thermal events: glass transition (T_g_ = −98 °C), sample melting (T_m_ = −10 °C), and the boiling of membrane components (T_b_ = 98 °C). For better visualization of the glass transition phenomenon, the temperature range from −125 °C to 0 °C was magnified and presented in panel B of Figure 5B.

Taking into account the fact that the individual components of the membranes are characterized by the presence of a glass transition at the following temperatures, T_g water_ = −135 °C, T_g PG_ = −68 °C, and T_g PVA_ = 80 °C, respectively, it was found that the glass transitions recorded in the thermograms correspond to a binary mixture of water and propylene glycol. Additionally, it was found that PG lowers the melting point of water by 10 °C, while the glass transition from PVA is invisible, i.e., covered by the endothermic process of boiling water.

To investigate the thermal response of the samples after water evaporation, the cryogels were initially heated to 185 °C, then cooled to −130 °C, and subsequently reheated. Each thermogram (Figure S2 in ESI) revealed a single thermal process corresponding to the glass transition of the studied samples. T_g_ values ranged from 6 °C to 60 °C, depending on the cryogel preparation method (i.e., freezing temperature). This suggests the presence of residual water bound to PVA and propylene glycol that did not evaporate during heating. It proves that the freezing temperature has a significant influence on the degree of the packing of water molecules in the prepared membranes and suggests the presence of both free and bound water. This, in turn, has a direct impact on the functional properties of the cryogels, which was discussed earlier.

The T_g_ values indicate that the least amount of water remained in the cryogel obtained by freezing at T = −80 °C (T_g_ = 60 °C). In contrast, the cryogels formed at −25 °C exhibited higher residual water content (T_g_ = 6 °C). These findings suggest that water evaporation is more challenging in materials formed at −25 °C or −196 °C compared to those prepared at −80 °C or −78 °C. This is consistent with microscopic studies (Figure 2), which showed that the cryogel formed at −80 °C had the largest pores. These larger pores likely facilitated more efficient water evaporation, contributing to the lower residual water content observed in this sample.

To assess the effect of the preparation method of hydrogel membranes on the amount and rate of water evaporation from them, a series of calorimetric studies were carried out involving the cyclic heating and cooling of the prepared cryogels. After each heating step to 70 °C, where the sample was held for 5 min (15 min for the final measurement), the sample was weighed, cooled to −130 °C, and subsequently reheated. Testing continued until no further changes in thermal response or sample mass were observed upon further heating. As can be seen in Figure S3 from ESI with subsequent cycles of cooling and heating of the samples, a decrease in intensity and a shift towards the lower temperatures of the process, reflecting the melting of the water–propylene glycol mixture, were observed. These changes, accompanied by a significant decrease in sample weight, confirm the phenomenon of water evaporation from the membranes. The complete disappearance of the melting process was observed after 20 min of annealing at 70 °C for membranes obtained by treatment with liquid nitrogen, 25 min for those treated with dry ice, 30 min for those frozen at −80 °C, and 60 min for those frozen at −25 °C.

Despite the lack of observed further evaporation at 70 °C, a residual fraction of unevaporated water remained in the cryogels, potentially evaporable at higher temperatures. The residual water percentages, determined through cryogel weight analysis (comparing initial and post-evaporation weights), were as follows: 18.9% for the cryogel formed at −196 °C (liquid nitrogen), 16.6% for the cryogel formed at −78 °C (dry ice), 17.1% for the cryogel frozen at −80 °C (low-temperature freezer), and 12.2% for the cryogel frozen at −25 °C (laboratory freezer).

**Figure 6:**
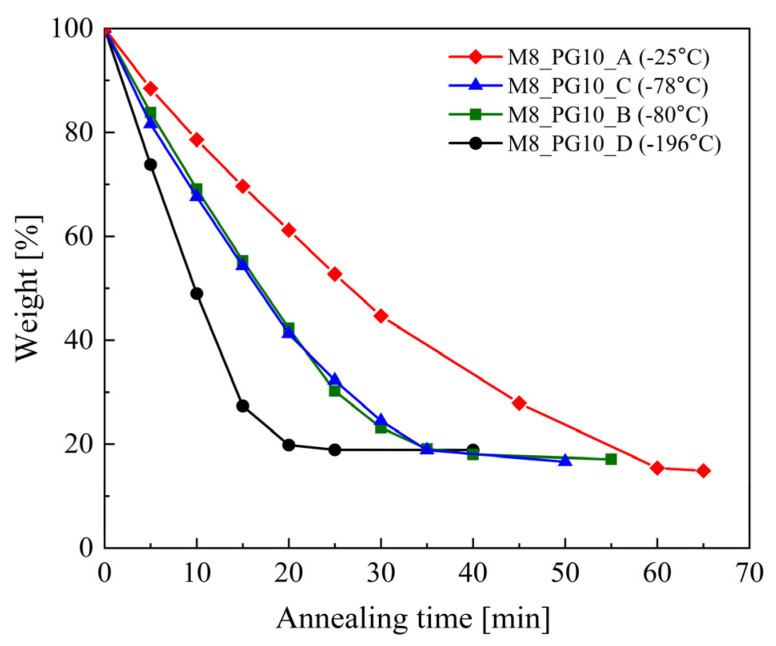
Time-dependent weight loss of M8 samples during cooling and heating cycles.

The differences in the rate of water evaporation from cryogels prepared in various temperatures are shown in Figure 6. The water in cryogel formed by freezing the PVA solution with liquid nitrogen evaporates the fastest. The intermediate water evaporation time was observed from the systems, prepared using dry ice and the −80 °C method. Meanwhile, the samples frozen at −25 °C showed the slowest rate of water evaporation. Notably, despite the rapid water evaporation observed in samples prepared by freezing the PVA solution with liquid nitrogen, a significant amount of water remained within the system. This suggests that rapid freezing forms a dense structure that hinders water escape. Moreover, rapid freezing may promote the formation of surface-bound or loosely bound water molecules within the matrix, which may not achieve full integration with the polymer network and are thus more readily released during the initial heating stages. However, further investigation is necessary to fully elucidate the underlying mechanisms of this behavior.

#### 3.3.2. NMR Analysis

The DSC studies were complemented by NMR relaxometry. Four samples were analyzed: two with 5% (*w*/*w*) PVA and two with 8% (*w*/*w*) PVA, all containing 10% (*w*/*w*) PG. These samples were prepared by freezing PVA_56–98_ solutions at two different temperatures: −25 °C (M5_PG10_A, M8_PG10_A) or −80 °C (M5_PG10_B, M8_PG10_B). This specific selection aimed to provide insights into the physical crosslinking process of PVA and assess the feasibility of these processes in hydrogel dressing technology.

In order to determine the transverse relaxation time T_2_ for the liquid signal, measurements of cryogels were performed using the CPMG sequence. The echoes of the transverse component decaying in time were fitted with the sum of two exponential functions according to Formula (3).

(3) $$f\left(t\right)=y_{0}+\sum_{i=1}^{3}A_{i}e^{-\frac{t}{{T_{2}}_{i}}}$$

where y_0_ is the constant level, A is the amplitude, and T_2_ is the spin–spin relaxation time.

**Figure 7:**
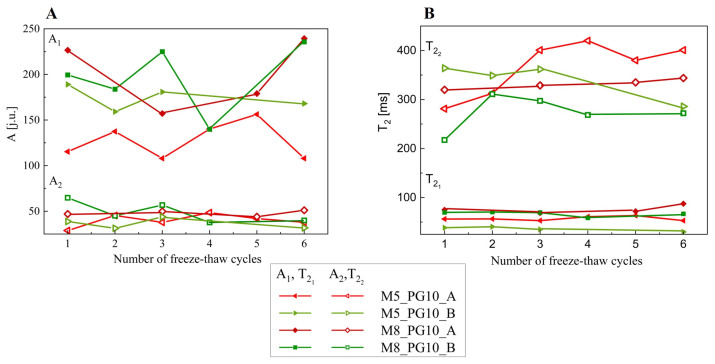
Graph of the dependence of parameters obtained from fitting function (3) to the data using the CPMG method, (**A**) amplitudes, and (**B**) relaxation times depending on the cycle.

The relaxation time parameters (T_21_ and T_22_) and amplitudes (A_1_, A_2_) are summarized in Table 6 and Figure 7. The amplitude values allowed for the estimation of proton density/concentration in each sample. The analysis of relaxation times provided insights into proton mobility and their binding or distribution within the polymer matrix.

In all analyzed cryogels, two distinct groups of protons with different T_2_ relaxation times were identified, indicating the presence of both bound and free water in varying proportions depending on the sample. The first group of protons was characterized by a short relaxation time T_21_ with high signal intensity A_1_, while the second one by longer T_22_ with low amplitude A_2_. By comparing the values of T_2_ relaxation times and taking into account the qualitative and quantitative composition of the analyzed cryogels, as well as their cross-linked nature, it can be concluded that the shorter component T_21_ is responsible for less mobile protons bound in the three-dimensional polymer network, while the longer component T_22_ reflects more mobile molecules, e.g., distributed between the chains of the polymer network (these are mainly free water protons mixed with propylene glycol, therefore their relaxation time is an order of magnitude higher). The data showed that both polymer concentration and freezing temperature had a significant impact on the physical crosslinking of PVA, influencing the properties of the cryogels. Freezing at −25 °C, regardless of PVA concentration, resulted in longer relaxation times (T_21_ and T_22_), indicating greater proton mobility due to a looser polymer network. This looser structure was associated with reduced mechanical strength, as confirmed by tensile testing (Table S2). In contrast, freezing at −80 °C resulted in shorter relaxation times, suggesting tighter polymer chain packing and reduced molecular mobility. All membranes exhibited higher A_1_ amplitudes than A_2_, indicating a predominance of bound water over free water, as expected from the physical crosslinking induced by freeze–thaw cycles.

In sample M5_P10_A, the lowest A_1_ amplitudes and the longest T_22_ (400.76 ms) were observed, indicating an inability of the polymer network to hold free water, which likely hindered the transition from a semi-solid to a solid sheet form (Figure 1). In sample M5_P10_B, shorter relaxation times and a higher A_1_ amplitude suggested the formation of a stiffer structure with more bound water compared to the same sample frozen at −25 °C. Considering the M8 samples frozen at temperatures of −25 °C and −80 °C, having very similar amplitudes and ratios, it was also noticed that a lower freezing temperature results in the formation of a stiffer network (greater cross-linking). The M5 membranes exhibited a looser structure and lower stiffness than M8, regardless of freezing temperature, consistent with mechanical property measurements and surface morphology analysis. Higher polymer concentration increased the amount of bound water within the membrane and reduced loosely bound water mixed with propylene glycol, as evidenced by both the amplitude total signal ratios and the higher T_21_ and lower T_22_ values for M8 samples compared to M5 samples.

These results were consistent with the DSC and SEM data. The smaller amount of loosely bound water in samples frozen at lower temperatures resulted in faster initial evaporation. However, due to the difficulty of removing water bound within the polymer chains, samples frozen at −80 °C ultimately retained more unevaporated water (see Figure 6). CPMG studies were complemented by SEM images, confirming that pores were better separated in samples prepared at −80 °C.

**Figure 8:**
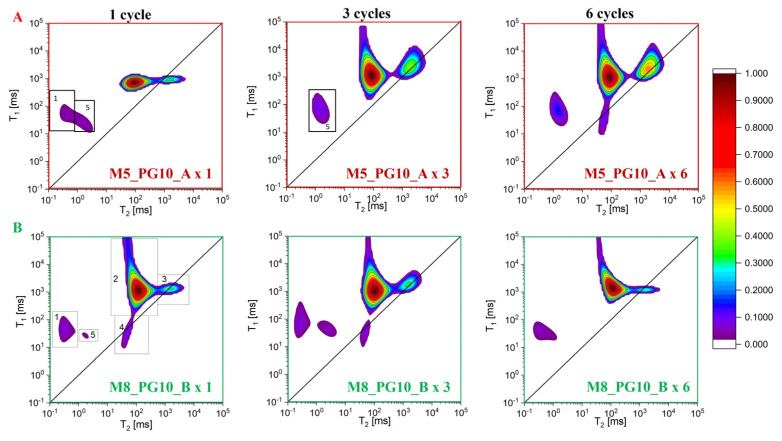
T_1_–T_2_ maps for selected hydrogel membranes obtained by cyclic freezing and thawing. (**A**) Freezing temperature of −25 °C; (**B**) Freezing temperature of −80 °C.

Two-dimensional relaxometry maps showed the distribution of free water (along the T_1_/T_2_ diagonal) and other proton groups of different mobilities, giving a more complex system description [46,47]. Two-dimensional maps allow the simultaneous measurement of both transverse (T_2_) and longitudinal (T_1_) relaxation times and combine the results in one diagram with T_2_ and T_1_ on diagram axes (T_1_–T_2_ maps), which is an extension of the CPMG method. The proportions, shape, and position of peak centers in the 2D maps varied depending on the freeze–thaw cycle, confirming the presence of free water mixed with propylene glycol in the membranes (peaks along the T_1_/T_2_ = 1 diagonal) and the dominance of loosely bound water within the polymer chains (the most intense peak at T_2_~100 ms and T_1_/T_2_~10). Each 2D map (Figure 8) revealed three primary peaks, with up to two additional peaks corresponding to groups of protons of different mobility. Peak 1, with T_2_ times below 1 ms (T_1_/T_2_~100), associated with less mobile protons in the polymer network, probably mainly originated from PVA. Peak 2, with the highest intensity and T_2_~100 ms (T_1_/T_2_~10), related to loosely bound water protons—its share in the total signal ranges from 65% for M5 samples to 88% for M8 (it was equivalent to the shorter component obtained from CPMG). Peak 3, closest to the diagonal (T_1_/T_2_~1) with T_2_~1 s, represents protons with mobility similar to free water (the equivalent of the long component in CPMG measurements), i.e., for free protons with the addition of PG, which causes the relaxation times to be longer. Peak 4, observed on some maps, suggests a pool of free water in small pores, with lower relaxation times than Peak 3 (T_2_~100 ms), i.e., more spatially limited (its T_2_ was an order of magnitude lower than in the case of Peak 3). The T_2_ times for Peak 4 (22–50 ms) and Peak 2 overlap in CPMG results, combining into a single short component with average T_2_ times of 30 to 88 ms. Hence, we obtain shorter T_2_ times for the component with higher intensity in the case of CPMG measurements than in the case of T_1_–T_2_ maps. Peak 5 denotes the proton pool of mobilized polymer (T_2_~1 ms and T_1_/T_2_ between 10 and 100). In M5 samples, the proton pool denoted as Peak 1 changed properties and moved to the position of Peak 5 (slightly mobilized PVA protons). In M8_PG10_B, Peaks 5 and 1 overlap (as in the case of M5 after one cycle).

## 4. Conclusions

To develop effective and comfortable wound dressings for clinical use, the designed materials must demonstrate essential properties such as structural integrity in contact with exudate, low adhesion, slightly acidic pH, high elasticity, and sufficient mechanical strength. Achieving these properties requires the careful selection of excipients and the optimization of manufacturing processes. Hydrogels produced using the freeze–thaw method have shown significant potential for wound management applications. However, their production requires precise control over critical factors such as freezing temperature and matrix-forming excipients, with no consensus on the optimal conditions for these variables. This study aimed to address this gap by investigating the impact of PVA grades and freezing conditions on cryogel performance, with a specific focus on their suitability for wound care applications.

The results indicated that fully hydrolyzed PVA with molecular weights exceeding 61,000 g/mol is critical for successful cross-linking and the formation of solid sheets through the freeze–thaw method. PVAs with lower molecular weights or partial hydrolysis were unsuitable for producing solid sheets. Among the PVA grades tested, PVA_56–98_ (Mw~195,000; DH = 98.0–98.8%) exhibited the most favorable properties in use as wound dressings, offering an optimal balance of elasticity, low adhesion, durability, and shape-memory properties. Despite its low water absorption capacity, it may still be suitable for dry or minimally exudative wounds that do not require materials with high absorbency.

Furthermore, while many studies commonly use −25 °C as the standard freezing temperature for cryogel preparation, we found that freezing the gel precursor at −80 °C offers superior results, producing materials with open, interconnected structures with improved mechanical strength and elasticity. This represents a notable deviation from conventional practices in cryogel production.

The DSC and NMR results confirmed the effectiveness of the cyclic freeze–thaw method in crosslinking the PVA solution, demonstrating a higher amount of bound water compared to free water in all cryogels, regardless of freezing temperature (A_1_ > A_2_ amplitude values in HMR). However, the bound water content varied between samples, indicating that freezing conditions influenced polymer chain packing. Thermal studies also showed that lower freezing temperatures retained more bound water in the samples, which is harder to evaporate, resulting in higher non-evaporated water content. For instance, 17.1% of water remained in the membrane frozen at −80 °C (M8_B), while 12.2% remained in the membrane frozen at −25 °C (M8_A), despite faster initial evaporation in M8_B due to larger pores, as confirmed by SEM. Shorter relaxation times (T_21_, T_22_) in samples frozen at lower temperatures reflect reduced proton mobility and suggest tighter polymer packing. This explains the higher mechanical strength and lower elasticity of cryogels formed at lower temperatures, as observed in tensile tests using a mechanical testing machine.

The cryogel prototypes developed in this study demonstrated functional properties comparable to, and in some respects exceeding, those of commercial wound dressings. However, their absorption capacity remained lower. Notably, the prototypes achieved high mechanical strength without the incorporation of additional reinforcing materials, which are commonly associated with a significant reduction in elasticity. The dressings exhibited the ability to deform and stretch up to 300% without mechanical rupture, offering enhanced comfort during application and removal. Despite their reduced absorption capacity, these findings suggest that the cryogel prototypes are promising candidates for wound care applications, particularly for the management of dry or minimally exuding wounds.

All in all, our findings underscore the importance of controlling freezing conditions and polymer characteristics to optimize hydrogel properties. They provide a foundation for the future development of advanced wound dressing systems, building on the progress achieved in this study.

## Figures and Tables

**Table 1:** Characteristics of different grades of polyvinyl alcohol (PVA), Mowiol^®^ (M).

No.	Type of PVA	Mw ^1^ [g/mol]	DH ^2^ [%]	Pw ^3^	Reference
1	PVA_8–88_	~67,000	86.7–88.7	~1400	[20]
2	PVA_18–88_	~130,000	86.7–88.7	~2700	[21]
3	PVA_10–98_	~61,000	98.0–98.8	~1400	[22]
4	PVA_20–98_	~125,000	98.0–98.8	~2800	[23]
5	PVA_28–99_	~145,000	99.0–99.8	~3300	[24]
6	PVA_56–98_	~195,000	98.0–98.8	~4300	[25]

^1^ Mw = molecular weight; ^2^ DH = degree of hydrolysis; ^3^ Pw = degree of polymerization.

**Table 2:** Composition of cryogels (% *w*/*w*).

Formulation Name	PVA ^1^ (% *w*/*w*)	PG ^2^ (% *w*/*w*)	W ^3^ (% *w*/*w*)
* M5_PG5	5.0	5.0	90.0
**M5_PG8**	5.0	8.0	87.0
**M5_PG10**	5.0	10.0	85.0
**M8_PG5**	8.0	5.0	87.0
**M8_PG8**	8.0	8.0	84.0
**M8_PG10**	8.0	10.0	82.0
**M10_PG5**	10.0	5.0	85.0
**M10_PG8**	10.0	8.0	82.0
**M10_PG10**	10.0	10.0	80.0

^1^ PVA, poly(vinyl alcohol); ^2^ PG, propylene glycol; ^3^ W, distilled water. * M, Mowiol^®^ (trade name of poly(vinyl alcohol).

**Table 3:** The values of pH, mucoadhesive properties (F_A_), and water uptake (U_w_) of polyvinyl alcohol solutions (10% *w*/*w*) and cryogels formulated with different PVA types (*n* = 6, ± SD).

PVA Type	Ph Value	F_A_ [N]	U_w_ [%]
PVA_8–88_	5.76 ± 0.10	Not tested ^1^	Not tested ^1^
PVA_18–88_	5.53 ± 0.07	Not tested ^1^	Not tested ^1^
PVA_10–98_	5.39 ± 0.12	Disintegrated ^2^	Disintegrated ^2^
PVA_20–98_	5.26 ± 0.21	0.220 ± 0.024	81.53 ± 4.03
PVA_28–99_	6.23 ± 0.20	0.135 ± 0.060	10.18 ± 0.95
PVA_56–98_	5.41 ± 0.17	0.099 ± 0.033	69.10 ± 4.13

^1^ Samples could not be tested due to the inability to achieve a solid sheet form as targeted in this study. ^2^ Samples did not maintain structural integrity, preventing the measurement.

**Table 4:** Mechanical properties of cryogels formulated with different PVA types (*n* = 6, ±SD) frozen at −80 °C.

PVA Type	Thickness [mm]	* σ [MPa]	* F_max_ [N]	* E [MPa]	* ε [%]	* e [%]
**PVA_20–98_**	3.19 ± 0.16	0.04 ± 0.01	0.54 ± 0.07	0.02 ± 0.00	265.44 ± 12.72	0
**PVA_28–99_**	3.32 ± 0.22	0.19 ± 0.02	3.47 ± 0.42	0.12 ± 0.01	238.98 ± 24.13	0
**PVA_56–98_**	3.29 ± 0.11	0.11 ± 0.02	2.08 ± 0.36	0.03 ± 0.00	348.97 ± 24.73	0

* Ultimate tensile strength (σ), breaking force (Fmax), Young’s modulus (E), maximum elongation at break (ɛ), and permanent deformation (e), respectively, determined by a tensile testing machine.

**Table 5:** Measured pore sizes and calculated porosity of xerogels formed at −80 °C (mean value ± SD).

Formulation Name	Parameters		
	Average Pore Width ^1^ [µm]	Average Pore Length ^1^ [µm]	Porosity ^2^ [%]
M5_PG10_B *	13.76 ± 9.29	28.35 ± 16.81	49.11 ± 2.46
M8_PG10_B *	10.79 ± 4.88	13.23 ± 8.20	46.94 ± 6.59
M10_PG10_B *	1.48 ± 0.38	4.42 ± 2.01	29.64 ± 3.30

* B-low-temperature freezer (temp. −80 °C). ^1^ *n* = 150. ^2^ *n* = 3.

**Table 6:** Relaxation time (T_21_, T_22_) and amplitudes (A_1_, A_2_) parameters for cryogels with 5% (*w*/*w*) and 8% (*w*/*w*) PVA content prepared by six-fold freezing at two different temperatures.

Formulation Name	Parameters			
	A_1_ [a.u.]	A_2_ [a.u.]	T_21_ [ms]	T_22_ [ms]
M5_PG10_A	107.97	37.82	53.07	400.76
M5_PG10_B	168.02	31.84	29.89	285.93
M8_PG10_A	239.36	51.03	87.47	343.87
M8_PG10_B	235.68	39.88	66.80	272.01

A refers to samples formed by freezing the PVA_56–98_ solution at −25 °C, while B refers to samples formed by freezing the PVA_56–98_ solution at −80 °C.

## Data Availability

Data are contained within the article and Supplementary Materials.

## Supplementary Materials

The following supporting information can be downloaded at: https://www.mdpi.com/article/10.3390/pharmaceutics16111388/s1. Figure S1: Visual appearance of cryogels prepared from six different grades of polyvinyl alcohol (PVA) at a 10% (*w*/*w*) concentration. Figure S2: Thermograms of cryogels after reheating post-water evaporation. Figure S3: DSC thermograms demonstrating rates of water evaporation from cryogels formed at various temperatures. Table S1: The values of pH, mucoadhesive properties (FA), and maximum water uptake (Uw) of solutions and cryogels formulated with the selected PVA grade (Mw~195,000; DH = 98.0–98.8%) (*n* = 6, ±SD). Table S2: Mechanical and physical properties of cryogels based on PVA_56–98_ under different freezing conditions (*n* = 6, ±SD). Table S3: Differences in ultimate tensile strength (σ) for the formulations. Table S4: Differences in breaking force (Fmax) for the formulations. Table S5: Differences in Young’s modulus (E) for the formulations. Table S6: Differences in maximum elongation at break (ε) for the formulations.
