# Supplementary material for: Physically Cross-Linked PVA Hydrogels as Potential Wound Dressings: How Freezing Conditions and Formulation Composition Define Cryogel Structure and Performance
Source: Pharmaceutics. 2024 Oct 28;16(11):1388. doi: 10.3390/pharmaceutics16111388 (PMC11597501; doi:10.3390/pharmaceutics16111388)
Supplement: Supplementary file 1 [file pharmaceutics-16-01388-s001.zip › pharmaceutics-3252533-supplementary.pdf]

Supplementary Materials

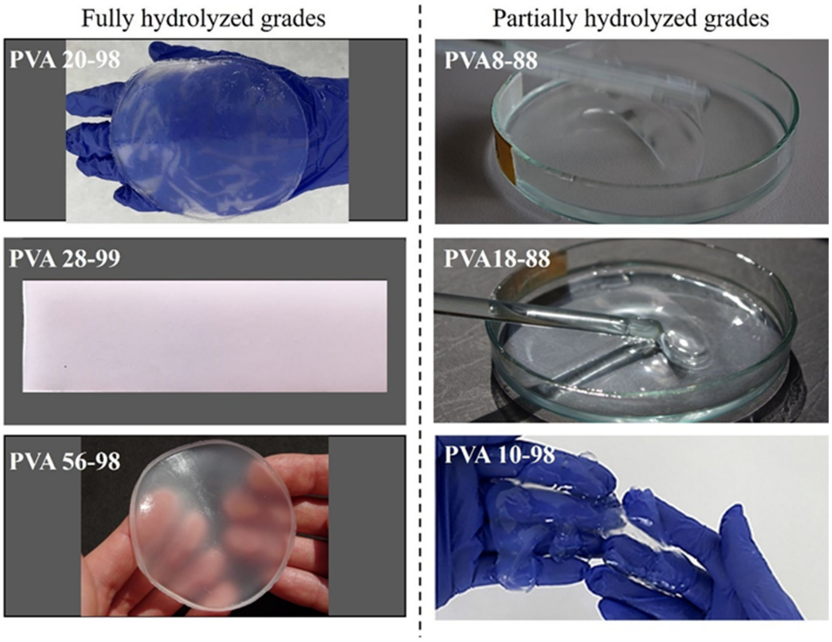

Figure S1. Visual appearance of cryogels prepared from six different grades of polyvinyl alcohol (PVA) at a 10% (w/w) concentration.

Table S1. The values of pH, mucoadhesive properties (FA), and maximum water uptake (Uw) of solutions and cryogels formulated with the selected PVA grade (Mw ~ 195,000; DH = 98.0-98.8%) (n = 6, ± SD).

Formulation name	Uw [%]	FA [N]	pH
**M5_PG5_A**	Not tested*		
**M5_PG5_B**	53.79 ± 4.31	0.062 ± 0.019	5.85 ± 0.28
**M5_PG5_C**	37.28 ± 5.43	0.056 ± 0.006	
**M5_PG5_D**	-	-	
**M5_PG8_A**	Not tested*		
**M5_PG8_B**	48.5 ± 23.0	0.055 ± 0.006	5.67 ± 0.25
**M5_PG8_C**	38.51 ± 10.47	-	
**M5_PG8_D**	-	-	
**M5_PG10_A**	Not tested*		
**M5_PG10_B**	46.7 ± 2.8	0.042 ± 0.009	5.62 ± 0.21
**M5_PG10_C**	34.07 ± 1.08	0.053 ± 0.006	
**M5_PG10_D**	-	-	
**M8_PG5_A**	62.52 ± 5.51	0.051 ± 0.025	
**M8 _PG5_B**	76.51 ± 7.77	0.032 ± 0.007	5.77 ± 0.44
**M8_PG5_C**	75.13 ± 22.14	0.045 ± 0.012	
**M8_PG5_D**	63.54 ± 3.29	-	
**M8_PG8_A**	57.90 ± 8.22	0.116 ± 0.026	
**M8_PG8_B**	70.23 ± 6.84	0.032 ± 0.003	5.47 ± 0.11
**M8_PG8_C**	91.19 ±7.20	0.044 ± 0.003	
**M8_PG8_D**	37.78 ± 1.19	-	
**M8_ PG10_A**	64.72 ± 16.51	0.076 ± 0.007	

**M8_ PG10_B**	81.80 ± 7.13	0.069 ± 0.008	5.47 ± 0,13
**M8_ PG10_C**	104.51 ± 6.69	0.102 ± 0.005	
**M8_ PG10_D**	44.50 ± 0.33	0.069 ± 0.008	
**M10_PG5_A**	58.1 ± 14.2	0.097 ± 0.024	
**M10_PG5_B**	68.27 ± 21.75	0.086 ± 0.032	5.56 ± 0.02
**M10_PG5_C**	48.22 ± 9.93	0.072 ± 0.019	
**M10_PG5_D**	32.12 ± 7.03	0.133 ± 0.063	
**M10_PG8_A**	80.96 ± 6.15	0.124 ± 0.020	
**M10_PG8_B**	62.39 ± 12.54	0.095 ± 0.030	5.54 ± 0.07
**M10_PG8_C**	71.34 ± 33.07	-	
**M10_PG8_D**	-	-	
**M10_PG10_A**	85.57 ± 7.63	0.089 ± 0.011	
**M10_PG10_B**	69.10 ± 4.13	0.099 ± 0.033	5.41 ± 0.17
**M10_PG10_C**	53.06 ± 10.51	0.089 ± 0.011	
**M10_PG10_D**	41.07 ± 5.21	0.091 ± 0.016	
**Product 1**	137.25 ± 9.25	0.045 ± 0.009	-
**Product 2**	180.29 ± 40.98	0.191 ± 0.035	-

* Samples could not be tested due to the inability to achieve a solid sheet form as targeted in this study.

A—laboratory freezer (temp. -25°C); B—low-temperature freezer (temp. -80°C); C—dry ice (temp. -78°C); D—liquid nitrogen (temp.-196°C).

Table S2. Mechanical and physical properties of cryogels based on PVA_{56-98} under different freezing conditions (n = 6, ± SD).

Formulation name	Mechanical strength		Flexibility	
	**σ [MPa]**	**Fmax [N]**	**E [MPa]**	**ε [%]**
M5_PG5_A	Not tested*			
M5_PG5_B	0.012 ± 0002	0.19 ± 0.03	0.009 ± 0.002	187.44 ± 23.98
M5_PG5_C	0.016 ± 0.06	0.27 ± 0.07	0.017 ± 0.005	175.29 ± 11.59
M5_PG8_A	Not tested*			
M5_PG8_B	0.009 ± 0.001	0.14 ± 0.02	0.007 ± 0.001	237.41 ± 10.93
M5_PG8_C	0.007 ± 0.001	0.11 ± 0.01	0.007 ± 0.000	157.36 ± 22.60
M5_PG10_A	Not tested*			
M5_PG10_B	0.009 ± 0.000	0.13 ± 0.01	0.004 ± 0.000	244.13 ± 32.77
M5_PG10_C	0.008 ± 0.002	0.12 ± 0.04	0.006 ± 0.001	216.41 ± 29.02
M8_PG5_A	0.048 ± 0.009	0.81 ± 0.17	0.022 ± 0.010	302.26 ± 18.62
M8 _PG5_B	0.044 ± 0.005	0.71 ± 0.11	0.025 ± 0.002	226.96 ± 24.05
M8_PG5_C	0.041 ± 0.008	0.67 ± 0.12	0.030 ± 0.002	205.14 ± 47.85
M8_PG5_D	0.056 ± 0.007	0.81 ± 0.06	0.035 ± 0.003	274.13 ± 18.42
M8_PG8_A	0.012 ± 0.002	0.19 ± 0.03	0.009 ± 0.002	231.43 ± 73.10
M8_PG8_B	0.110 ± 0.030	2.33 ± 0.55	0.070 ± 0.090	226.17 ± 38.91
M8_PG8_C	0.074 ± 0.008	2.63 ± 0.50	0.074 ± 0.008	253.12 ± 13.36
M8_ PG10_A	0.020 ± 0.000	0.40 ± 0.06	0.009 ± 0.001	306.09 ± 22.76
M8_ PG10_B	0.062 ± 0.011	1.11 ± 0.21	0.025 ± 0.004	349.63 ± 20.59
M8_ PG10_C	0.061 ± 0.006	1.01 ± 0.12	0.038 ± 0.003	242.29 ± 23.71
M8_ PG10_D	0.080 ± 0.019	1.34 ± 0.36	0.053 ± 0.007	222.03 ± 31.53
M10_PG5_A	0.027 ± 0.005	0.45 ± 0.08	0.018 ± 0.005	234.33 ± 18.66
M10_PG5_B	0.054 ± 0.004	0.89 ± 0.05	0.038 ± 0.003	193.22 ± 30.58
M10_PG5_C	0.130 ± 0.015	2.16 ± 0.29	0.080 ± 0.004	205.44 ± 23.98

M10_PG5_D	0.107 ± 0.033	1.70 ± 0.53	0.067 ± 0.016	256.78 ± 35.41
M10_PG8_A	0.025 ± 0.008	0.41 ± 0.17	0.016 ± 0.002	208.93 ± 44.81
M10_PG8_B	0.137 ± 0.004	2.29 ± 0.07	0.084 ± 0.001	239.76 ± 9.06
M10_PG8_C	0.149 ± 0.011	2.53 ± 0.26	0.099 ± 0.004	256.5 ± 14.87
M10_PG8_D	0.092 ± 0.000	1.37 ± 0.05	0.086 ± 0.002	187.87 ± 14.14
M10_PG10_A	0.074 ± 0.008	1.24 ± 0.14	0.026 ± 0.003	287.56 ± 20.29
M10_PG10_B	0.105 ± 0.015	2.08 ± 0.36	0.027 ± 0.003	348.97 ± 24.73
M10_PG10_C	0.136 ± 0.038	2.29 ± 0.62	0.081 ± 0.021	267.26 ± 24.13
M10_PG10_D	0.105 ± 0.004	1.63 ± 0.02	0.074 ± 0.013	249.23 ± 9.64
Product 1	0.004 ± 0.001	0.08 ± 0.02	0.004 ± 0.002	85.04 ± 18.34
Product 2	0.330 ± 0.082	3.64 ± 0.43	0.146 ± 0.024	290.29 ± 26.38

* Samples could not be tested due to the inability to achieve a solid sheet form as targeted in this study.

A—laboratory freezer (temp. -25°C); B—low-temperature freezer (temp. -80°C); C—dry ice (-78°C); D—liquid nitrogen (-196°C). Ultimate tensile strength (σ), breaking force (F_{max}), Young's modulus (E), and maximum elongation at break (ε), respectively, determined by a tensile testing machine.

Table S3. Differences in ultimate tensile strength (σ) for the formulations.

	M8_PG10_A	M8_PG10_B	M8_PG10_C	M8_PG10_D
M10_PG10_A	S	NS	S	S
M10_PG10_B	S	S	S	NS
M10_PG10_C	S	S	S	S
M10_PG10_D	S	S	S	NS

S—significant difference (p<0.05)

NS—non-significant

Table S4. Differences in breaking force (Fmax) for the formulations.

	M8_PG10_A	M8_PG10_B	M8_PG10_C	M8_PG10_D
M10_PG10_A	S	NS	NS	NS
M10_PG10_B	S	S	S	S
M10_PG10_C	S	S	S	S
M10_PG10_D	S	NS	S	NS

S—significant difference (p<0.05)

NS—non-significant

Table S5. Differences in Young's modulus (E) for the formulations.

	M8_PG10_A	M8_PG10_B	M8_PG10_C	M8_PG10_D
M10_PG10_A	NS	NS	NS	NS
M10_PG10_B	NS	NS	NS	NS
M10_PG10_C	S	S	S	NS
M10_PG10_D	S	S	NS	NS

S—significant difference (p<0.05)

NS—non-significant

Table S6. Differences in maximum elongation at break (ε) for the formulations.

	M8_PG10_A	M8_PG10_B	M8_PG10_C	M8_PG10_D
M10_PG10_A	NS	NS	NS	S
M10_PG10_B	NS	NS	S	S
M10_PG10_C	NS	S	NS	NS
M10_PG10_D	NS	S	NS	NS

S—significant difference ($p<0.05$)

NS—non-significant

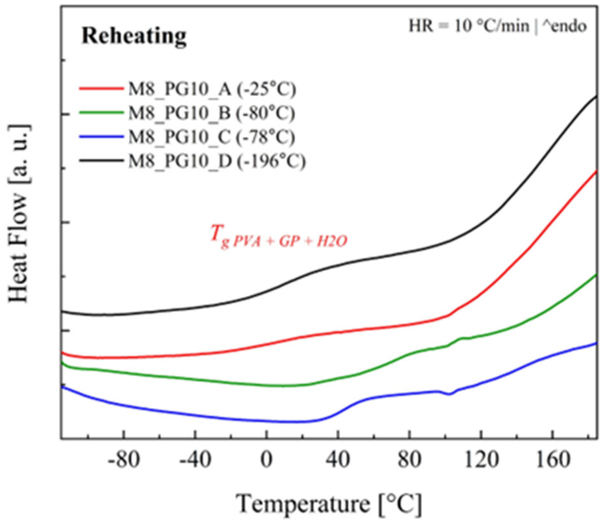

Figure S2. Thermograms of cryogels after reheating post-water evaporation.

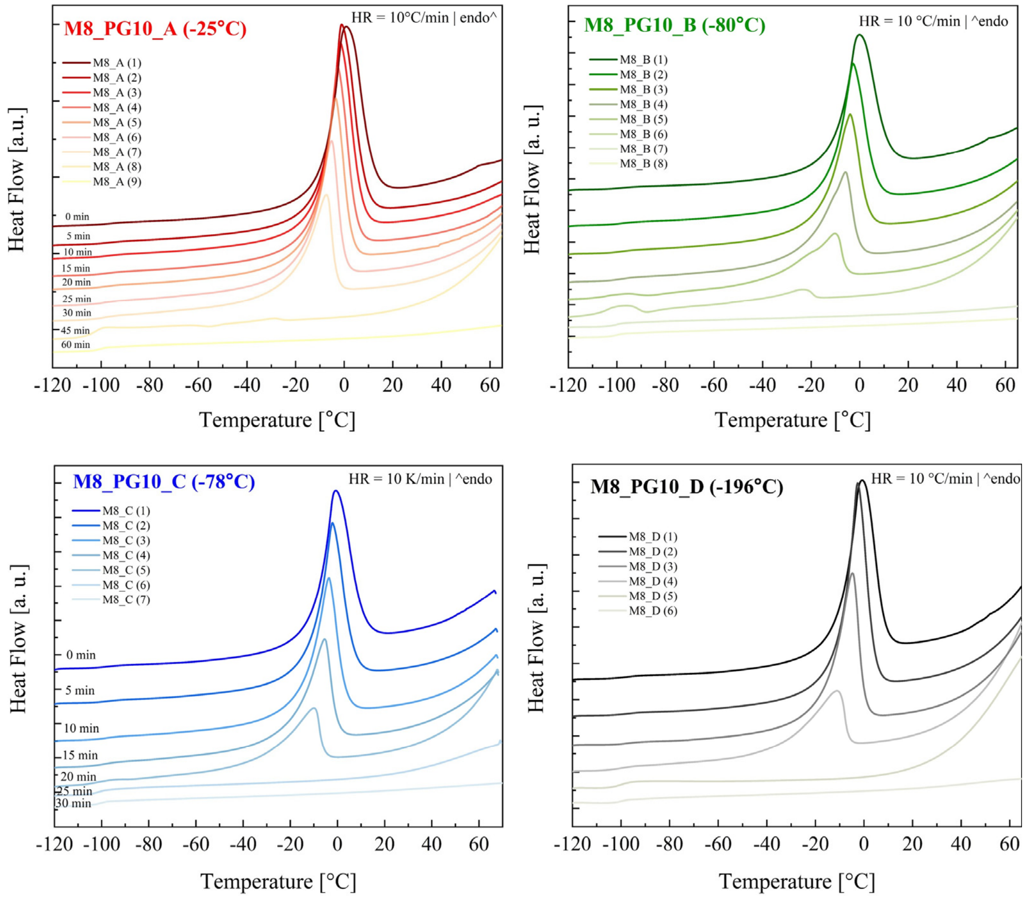

Figure S3. DSC thermograms demonstrating rates of water evaporation from cryogels formed at various temperatures.
